# Effect of nanostructure lipid carrier of methylene blue and monoterpenes as enzymes inhibitor for *Culex pipiens*

**DOI:** 10.1038/s41598-023-39385-y

**Published:** 2023-08-02

**Authors:** Ibrahim Taha Radwan, Mohamed Z. Sayed-Ahmed, Nirvina AbdelRaouf Ghazawy, Saad S. Alqahtani, Sarfaraz Ahmad, Nawazish Alam, Abeer Mousa Alkhaibari, Md Sajid Ali, Abdelfattah Selim, Eman Alaaeldin AbdelFattah

**Affiliations:** 1 Supplementary General Sciences Department, Faculty of Oral and Dental Medicine, Future University in Egypt, Cairo, 11835 Egypt; 2 Department of Clinical Pharmacy, College of Pharmacy, Jazan University, 45142 Jazan, Saudi Arabia; 3 Department of Internal Medicine and Infectious Diseases, Faculty of Veterinary Medicine, Mansoura University, Mansoura, 35516 Egypt; 4 Department of Entomology, Faculty of Science, Cairo University, Giza, Egypt; 5 Department of Clinical Pharmacy, College of Pharmacy, King Khalid University, Abha, Saudi Arabia; 6 Department of Biology, Faculty of Science, University of Tabuk, 71491 Tabuk, Saudi Arabia; 7 Department of Pharmaceutics, College of Pharmacy, Jazan University, Jazan, 45142 Kingdom of Saudi Arabia; 8 Department of Animal Medicine (Infectious Diseases), Faculty of Veterinary Medicine, Benha University, Toukh, 13736 Egypt

**Keywords:** Enzymes, Chemistry

## Abstract

Solid lipid nanoparticles second generation, nanostructure lipid carrier (NLC), is one of the most important biodegradable nanoparticles. Nanostructure Lipid carrier (NLC) was used to encapsulate methylene blue (MB) dye, carvacrol and citronellal and their efficacy as insecticidal against *Culex pipiens* (*Cx. pipiens*) were distinguished. The prepared nanoformulation revealed very good physicochemical properties, especially the homogeneity of the particle size. Transmission electron microscope showed spherical shaped nanoparticles within range less than 200 nm. The prepared NLC-MB-MT system showed a very competitive insecticidal activity and high virulence against the mosquito larvae with higher mortality rate of LC_50_ of 0.141 µl/mL, in addition to high level of Oxidative stress parameters obtained through all the tested enzymes including hydrogen peroxide (4.8 ppm), protein carbonyl amount (0.12 OD/mg protein), ascorbic acid (0.15 mg) and Superoxide dismutase (SOD) showed strong increasing (0.09 OD/mg protein/min) at 6 µg/mL, respectively. Whereas paradoxical results of the oxidative stress enzymes were obtained from different concentration of nanoformulation that introduce a convenient reason for their potential insecticidal effect. The cytotoxic effect of NLC-MB-MT was evaluated using WI38 human lung cell lines, the LC_50_ was 6.4 mg/mL. The low cytotoxic reactivity towards the tested cell line makes the NLC-MB-MT nanoformulation has its promising insecticidal efficacy. Molecular docking study for each component were done against acetylcholine esterase protein and accepted binding modes achieved by the three compounds.

## Introduction

Beginning in the 1990s, solid lipid nanoparticles (SLN) were developed as alternative to other carrier systems like emulsions, liposomes, and polymeric nanoparticles. Even though the advantages of SLNs are high drug protection^1^ from both chemical and enzymatic degradation^2^, physical stability, the ability to incorporate hydrophilic and hydrophobic drugs^3^, avoid organic solvents^4^, the ability to carry more than one drug (co-delivery)^5^, high drug entrapment efficiency small diameter and narrow size distribution^6^, administration via different pathways, bio degradable^5^, controlled drug release^7^, site specifying targeting^8^, biocompatibility^9^, increasing the drug bioavailability^10^, minimizing the side effects and its high ability to cross biological system barriers, but it has its special drawbacks that encouraged to substantial change in the SLN structure.

On another side, the disadvantages of SLN like the drug expulsion after polymeric transitions during storage, low drug loading capacities, agglomeration and polydispersity (wide range of particle size) that made it inappropriate for the intravenous administration^11^. Due to the mentioned drawbacks in addition to SLN tends to form perfect crystal structure because of the presence of solid lipid during the solidification process, the second-generation nanostructured lipid carrier (NLC) was devolved. The NLC distinguished with higher drug loading capacities and entrapment efficiency^12^, modulation of drug release pattern^13^, long-term stability of the encapsulated drug during storage in addition to their enhanced stability profile^13^.

Blocking the electron transport chain (ETC) complex is the main targets that several pesticides were designed, which targets the mitochondria via diminishing the efficacy of oxidative phosphorylation process. The hydramethylnon for example, inhibited complex 3^14^, fenazaquin, tolfenpyrad, and pyridaben inhibited complex 1^15,16^. Additionally, Fungicide Pristine^®^ inhibited the mitochondrial function in honeybees^17^, whereas some other types have dual activity like, Fipronil and imidacloprid influence multiple functional parameters of bee mitochondria and reduce the activity of these organelles^18^.

Organophosphorus pesticides commonly affect oxidative stress in addition to huge mitochondrial dysfunction^19^. Alternatively, Pyrethroids could cause various mitochondrial changes, deactivate formation of the mitochondrial membrane potential, enhance the production of reactive oxygen species (ROS), mutate the fluidity of lipid's mitochondrial membrane beside massive damage of the mitochondrial DNA^20^.

Searching for alternative and eco-friendly pesticides to achieve minimal human and environmental risks is crucial demand. Insecticides based on the Botanical extract become more famous as alternatives to the synthetic ones, which were confirmed to their insecticidal activity^21,22^. The majority of plant essential oils are made primarily of monoterpenes, that natural compounds give plants their distinct odoriferous characteristics. Due to their unique chemical and physical properties, make them suitable lead chemicals for the creation of entirely biodegradable and environmentally benign insecticides. Numerous investigations have been done on the ability of monoterpenes to repel and kill different types of mosquitoes^23^. Carvacrol, is a monoterpene phenol presented in many types of essential oils. It was known as one of the most potent natural essential oils that have great insecticidal effect against various types of insects especially *Cx. piepens* in addition to their repellent activity towards wide range of insects^24^. Citronellal, is the main constituent of citronella extract which gives the distinctive lemon aroma which is often used as strong insect repellent due to its very strong aroma. More studies were done to investigate Citronella preparations in preventing mosquito bites^25^. The conjugation of more than mono terpenes (like in extracts) at the same nanoformulation grantee additional insecticidal activity^26,27^.

Methylene blue dye (MB) is one of the thiazine dyes which has recently attracted the attention of researchers due to its newly discovered biological activities related to its structure and photosensitization efficacy. Light irradiation of mixture composed of Eosin-Methylene blue (EMB) was found to be extensively phototoxic to *Ae. aegypti larvae* at a very low concentration (0.5 µg/mL) EMB mixture presented a fast internalization time into the larvae, with rapid death induction efficient larval mortality using either sunlight or irradiation from a white-lighting source. The results furnished about 100% larval mortality when the larvae were exposed to sunlight for about 40 min, meanwhile no significant toxicity effect to the EMB mixture was recorded in the darkness^28^.

The *Cx. pipiens* complex of mosquitoes are significant disease carriers with a widespread distribution. They transmit the West Nile virus, the rift valley virus, the Bancroftian or lymphatic filariasis-causing *Wuchereria bancrofti*, the canine heartworm *Dirofilaria immitis*, and bird malarias (*Plasmodium relictum*)^29–35^.

Immune responses in mosquitoes include both humoral and cellular components. Production of anti-microbial peptides is part of the humoral process. Hemolytic cells, on the other hand, are part of the cellular response and play a role in a variety of responses, including phagocytosis and mosquito encapsulation. Oxidizing agents such as hydrogen peroxide (H_2_O_2_, a normal by-product of cellular metabolism in aerobic cells) are used as biocides in medical, food, and industrial applications due to their broad-spectrum activity, which includes efficacy against bacterial endospores, their lack of environmental toxicity, and their complete degradation over time^36^. These agents react with macromolecules, such as deoxyribonucleic acid (DNA), ribonucleic acid (RNA), proteins, and lipids, causing alterations to their structures^37^.

In insects, as in other animals, lipid peroxidation is potentially very harmful because lipids are components of cell membranes and play an important role in developmental and reproductive physiology^38^. Moreover, the high concentrations of reactive oxygen species (ROS) damage the absorption of ingested nutrients and can cause oxidative damage to the midgut cells and impair the absorption of ingested nutrients^39^. However, fragmentary reports are available on their overall physiological impacts on flies. In biological systems, oxidative stress occurs when the balance between ROS and antioxidants is disrupted because of excess ROS, depletion of antioxidants, or both^40,41^. To protect against the oxidative damage of ROS, insects possess specific defense mechanisms in the form of a network of antioxidant enzymes associated with the protection against oxidative stress^42,43^.

The enzymatic antioxidants include catalase (CAT), superoxide dismutase (SOD), Glutathione peroxides (GPx), and glutathione reductase (GR). Moreover, the non-enzymatic antioxidants include reduced glutathione (GSH), α-tocopherol, and β-carotene^38^. Macromolecules damage products and enzymatic antioxidants activity or non-enzymatic antioxidants can be used as indicators of oxidative stress^38,44^. Besides that, reducing power ability and total antioxidant ability can measure the antioxidant activities and act as a bio-chemical marker of oxidative stress^45,46^.

Acetylcholinesterase (AChE) it is a key enzyme in the Central Nervous System (CNS) of the insects specifically is the major inhibitory neurotransmitter in the insect central nervus system which terminates the nerve impulse by catalyzing the hydrolysis of the neurotransmitter acetyl choline. Most of insects possess two different types of AChE the first type is AChE and the second Pseudocholinesterase (BuChE) or plasma cholinesterase. Inhibition of AChE on the neuromuscular system that induces cholinergic crisis and all the CNS affected that makes targeting AChE inhibition is great demand. Using molecular docking and bioinformatics is very effective tool to help researchers to choose best inhibitor and also to shrink the probabilities instead of laboratory and field experimentation^47^.

The vitamin is present in numerous tissues where it likely functions in a number of ways that are connected to its redox potential. l-ascorbic acid may also play a role in metabolic processes like tyrosine metabolism, collagen creation, steroid synthesis, detoxification reactions, phagostimulation, or neuromodulation in addition to its potential general role in detoxifying superoxide (O_2_) and hydrogen peroxide (H_2_O_2_)^48,49^.

Therefore, the current study aimed to improve and understand the use of NLC incorporated with MB carvacrol and citronellal mono Terpenes (NLC-MB-MT) as insecticide via studying to oxidative stress parameters as key of protective indicators towards *Cx. pipiens* larvae and to evaluate: (i) ROS concentration (H_2_O_2_) (ii) determines enzymatic antioxidants activity (Ascorbic acid concentration& SOD activity) (iii) protein carbonyl amount after treatment by NLC-MB-MT with different concentrations on the fourth instars of *Cx. pipiens* with respect to control insects.

## Materials and methods

### Nanostructured lipid carrier (NLC)

Methylene blue (MB) dye 89%, Stearic acid 97%, oleic acid 90%, De-ionized water, polysorbate 20 (tween20), sodium glycocholate 97.5% and sodium tourocholate, 96% were purchased from Alfa Aesar (Thermo Fisher Scientific, Germany), while citronellal 93% and carvacrol 95% monoterpens were obtained from Acros Organics (Thermo Fisher Scientific, Germany). All the chemical reagents were used without any purification.

###  Synthesis of encapsulated nanostructure lipid carrier

The preparation of lipid carrier nanoparticles encapsulated MB, carvacrol and citronellal monoterpenes (NLC-MB-MT) was done by the assistance of the homogenization method according to Radwan et al.^50^. It was prepared by using three different beaker (B1), (B2) and (B3). Firstly, the aqueous solution prepared (B1), 4 mL de-ionized water, 3 mL Tween 20 and 0.2 mL butanol as co-surfactant were mixed to a well-stirred and pre-warmed mixture of 0.25 g sodium glycocholate and 0.25 g sodium taurocholate solubilized in 10 mL water then kept warmed at 45 °C.

On another beaker (B2), the lipid contents with the drugs need to be encapsulated, were placed with the quantities of 0.9 g of stearic acid, and 0.018 g of MB. A stock mixture of methanol and chloroform 3:1 (v/v) was prepared and 2 mL withdrawn from the solvent stock solution and used to dissolve MB, the temperature raised gradually up to 85 °C, consequently the stearic acid became molten and the MB dissolved, keeping the heating for 7 min further until all the solvent evaporated (use digital balance to make sure that all the weighted solvent evaporated and no effervescence due to solvent still present). Allow the beaker (B2) to cool up to 70 °C using infra-red thermometer to monitor the temperature change and keep heating to this temperature, the molten should be clear blue solution if there was any non-dissolved MB the dissolution step should be repeated with increasing the quantity of stearic acid.

Using 5 mL beaker (B3), exactly 1.2 g of oleic acid and 0.02 g of both carvacrol and citronellal (each one 0.01 g) were added. The mono terpenes (MT) was injected beneath the oleic acid layer using micropipette to reduce volatile ability, then the mixture stirred at 150 rpm for 2 min. The content of beaker (B3) was added to beaker (B2) and mixed together mechanically at 250 rpm at the same temperature for one min (the overheat avoided) quickly, the beaker (B1) was added to the mixture with stirring at 600 rpm for 2 min and the final mixture was quenched by adding 20 mL ice cold water and sonication applied using probe sonicator (VCX 750 sonicator & 13 mm probe) for 15 min then the final emulsion was kept in 50 mL falcon tube at a temperature 10 °C.

### Characterization of nanostructure lipid carrier

#### The average particle size (DLS), polydispearsity index (PDI) and zeta potential

The radius and polydispersity index (PDI) were measured by using the dynamic light scattering technique (DLS). The measuring conditions were set to be at 25 °C (the room temperature) with an angel of 173°. Zeta potential (z.p) was investigated by estimating the frequency shift change of the scattered light at a scattering angle of 12° due to laser beam irradiation. The average size, PDI and zeta potential measurements were done using (Zeta sizer Nano ZS, Malvern Instruments Ltd., Malvern, UK) in the Egyptian petroleum research institute (EPRI). The sample preparation by dissolving or dispersing about 5–10 mg of the solid NLC (if lyophilized) or the solution under investigation in 20 mL de-ionized water at the room temperature (25 °C) with homogenization (preferred to used probe sonication) for 5 min before investigating the sample, the sample investigated at zero day and 7-day preparation.

#### Internal morphology by transmission electron microscope (TEM)

The shape of the NLC prepared and the internal structure visualization was carried out using Field Transmission Microscopy (HR-TEM, JSM-7100F) in the central labs of National Research center institute (NRC), Giza, Egypt. The images were recorded with JEOL JEM-2100-115 high-resolution transmission electron microscopes system the electron- accelerating voltage varied from 100 to 200 kV. The NLC sample prepared as follow, 1 µL of NLC was highly diluted with de-ionized (1:200 v/v) and placed and fixed on a 200 mesh carbon-coated grid, after 2 min the excess of liquid NLC was removed by cellulose filter. Phosphotungstic acid (PTA) was dropped (2–3 drops) to the grid for 10 s to allow the negative staining to occur then the excess PTA was removed via filter paper by absorption.

### In-vitro cytotoxicity effect of the NLC-MB-MT nanoformulation on normal human cell lines (MTT assay)

The fibroblast human lung cell lines WI38 (Siga Aldrich, Merck, Darmstadt, Germany) was used to perform the cytotoxicity assay of the NLC-MB-MT nanoformulation (American type culture collection, CCl-75). The cell line cultured RPMI media containing 10% fetal bovine serum (FBS). Antibiotics 100 unit/mL were added, penicillin and streptomycin (each one 100 µg/ml to prevent any bacterial growth) and then incubated using CO_2_ incubator at 37 °C with humidity rate 5%. The cell lines were transported and seeded in 96-well plates at a density 1.0 × 10^5^ per well and incubated for 48 h after getting the desired cell-confluence, the cells treated with different concentrations (1000, 500, 250, 125, 62.5 and 31.25 µg/mL) of the NLC-MB-MT nanoformulation and re-incubated at the same conditions of temperature and humidity for 48 h. To determine the cell viability and consequently the IC_50_, 20 μL of MTT solution at 5 mg/mL was added for each seeded well and incubation processed for additional 4 h. The mitochondrial succinate dehydrogenase enzyme in viable cells will convert the yellow soluble tetrazolium salt into purple pellet of insoluble formazan compound, while the non-viable cells will not. The amount of purple formazan produced quantified calorimetrically after adding 100μL Dimethyl sulfoxide (DMSO) for each well to dissolve the produced formazan completely. Using a plate reader (EXL 800, USA), the colorimetric assay was measured at ƴ_max_ = 570 nm. The relative cell viability (%) was determined as (treated samples/ untreated sample) × 100. The values of cell viability converted into toxicity and consequently to the IC_50_.

### Molecular docking of acetylcholine esterase enzyme

#### Source of the objective protein

The binding affinity of MB, citronellal and carvacrol against the *Ls*-AChBP binding site were analyzed using theoretical approach, molecular docking, to determine whether the compounds make interaction in the binding site of the protein. There is no three dimensional crystal structure of acetylcholine esterase nAChR in the Protein dada bank, so that the well-known crystal three dimensional structure of the *Lymnaea stagnalis* acetylcholine binding protein *Ls*-AChBP (PDB Code: 2ZJU) was used in the docking study without homology modeling (Ihara, 2008), (El-Sayed, 2023) was downloaded from protein data bank (https://www.rcsb.org/structure/2ZJU). The protein downloaded as a form of PDB format, water molecules in addition to any heteroatom were removed, keeping only chain A.

#### Energy minimization

The tested compounds of MB, citronellal and carvacrol were drawn using the software CAMBRIDGESOFT CHEMOFFICE 2015 Professional 15.0.0 and memorized as Mol format followed by energy minimization using the default Amber12: EHT force field till gradient convergence of 0.01 kcal/mol was obtained by Molecular Operating Environment MOE_2015.10, which installed by 64-bit operating system [Intel (R) Core (TM) i5-2400 CPU @ 2.40 GHz, 8 GB RAM] system.

### Docking procedure

The reference drug or co-crystallized drug (imidacloprid) was labeled in green color to be distinguished easily, binding sites was recognized automatically form the surface sand maps options to recall the co-crystallized ligand binding site directly and the docking completed after ligands and protein preparation, using default setting were used via Rotate Bonds” choice to permit flexible ligand-rigid receptor docking. The score function was changed to be the London G with triangle matcher replacement were set, 30 conformer was adjusted instead of the automatic option conformer of the best score ligand and low energetically were retained. The top five conformers scoring of ligand-receptor docking was then showed by two- and three-dimensional ligand-receptor interactions^51,52^.

### Insecticidal evaluation

#### Insecticidal effect and enzyme assessment

A colony of *Cx. pipiens* larvae were supplied from Department of Entomology, Faculty of Science, Cairo University. The larvae were kept in a plastic plate 20 * 20 * 10 cm^3^ for 200 larvae in each plate. Larvae were supplied daily with fish food. The NLC-MB-MT was prepared by dissolving the different concentrations in 5 mL distilled water (1, 2, 4, 6 µL/mL) then adding larvae in each concentration. Control insects were treated with distilled water only.

The experiment was stand along 24 h. Then, the mosquito larvae were collected using mesh and then were homogenized in 5 mL of an ice-cold phosphate buffer with additions from (60 mL of 50 mM phosphate buffer, 10 mL of 0.1% Triton 100, 5 mL of 0.05 mM CaCl_2_; the mixture was filled with distilled water to a volume of 100 mL after correcting pH to 7.0 with HCl or NaOH). The samples were centrifuged at 2000×*g* for 10 min at 4 °C after homogenization (10 strokes/30 s using a pestle). Three replicates of the measurements were made (each replicate was a pool of ten insects). The Levine et al.^53^ method was used to calculate the amounts of protein carbonyls. The bovine serum albumin (BSA) fraction V (Sigma-Aldrich, Missouri, United States) was used as the protein standard to measure the total protein concentration of the samples using the Bradford^54^ technique by spectrophotometry.

The Misra and Fridovich^55^ technique was used to measure SOD activity. The reaction mixture looked like this: 87 mL of the appropriate tissue's supernatant, 402 mL of a sodium carbonate buffer (200 mM; pH 10.0), 35 mL of EDTA (10 mM), and 2835 mL of newly produced epinephrine (15 mM). In order to quantify the absorbance, a UV/Vis Jenway-7305 spectrophotometer was used (Bibby Scientific Limited, Staffordshire, UK). OD/g protein/min was used to express SOD activity.

For Ascorbic acid determination, 10 g of the sample was blended in the blender, the sample extract is created. A 250 mL conical flask was then filled with the sample and 50 mL of a 5% metaphosphoric acid acetic acid solution. The flask was filled with the remaining 50 mL of phosphoric acid solution. After using Whatman filter paper to filter the solution, the filtrate was collected to be tested for vitamin C. A small amount of bromine solution was added and blended with the filtered sample solution. To eliminate the bromine solution, a few drops of thiourea solution were then added to the sample solution. The sample solution then mixed with 1 mL of a 2,4 DNPH solution. 2,4 DNPH solution causes the coupling process. All of the standards and sample solution were held at 37 °C for 3 h to allow the reaction to finish. 5 mL of H_2_SO_4_ was added after the solutions had cooled on an ice bath for three hours. Thus, colored solutions were produced, and their absorbance was measured at a certain wavelength^56^.

The method of Junglee et al.^57^ was used to determine the concentration of H_2_O_2_ spectrophotometrically. Simply put, a one-step extraction-colorimetric procedure was used, consisting of a homogenization step using PBS, pH = 7.0 mixed with 0.25 mL Trichloroacetic acid (TCA) (0.1% (w:v)), 0.5 mL KI (1 M), and then centrifuging the 1 mL samples at 12,000 xg for 15 min at 4 °C to measure the absorbance at 240 nm.

### Laboratory rearing of *Cx. pipiens*

*Cx. pipiens* was initially obtained from the Kerdasa area in the Giza Governorate and colonized at the laboratory of the Cairo University's Entomology Department (1985) after being morphologically identified on the taxonomic keys by Harbach and Knight^58^. Three hundred of newly born larvae were transferred into a deep covered white enamel pan have floating fish food (protein 48.0%, oil 8.0%, fibre 2.0%, ash 11.0%, and moisture 6.0%). To ensure best rearing conditions, the water lost due to evaporation was compensated with fresh distilled water and the dead larva were removed day by day. Using a collecting sieve, the pupae removed from the breeding pans into plastic pots (7 cm × 6 cm) half filled with de-ionized water then the pupae were transported to the adult breeding cages for adult emergence. Emerging adult *Cx. pipiens* mosquitoes were placed into a mesh-screened wooden cage of dimensions (35 × 35 × 35 cm) with a hole in the front for pupae insertion and the other daily-routine work of food insertion, removal of laid eggs, and cage cleaning. The cages were supplied with a small petri dish with a cotton pad soaked in a 10% sucrose sugar solution as a source of carbohydrates for both males and females. The sucrose solution was changed every day to prevent fungus contamination. To confirm blood feeding which was provided 3–4 days following the emergence of the female, a domestic pigeon was tied to the top of the rearing cage at the night shift, noticing that before the first blood meal the sugar was withheld for 12 h. A 250 mL plastic cups partly filled with dechlorinated tap water were used to allow the gravid females to oviposit while also increasing the relative humidity in the cage. Daily collections of oviposited egg rafts were made, and these were delicately transferred with a tiny paintbrush to white enamel pans (25 cm in diameter and 9 cm in depth), which were half filled with dechlorinated tap water. These pans were preserved until the eggs hatched and are covered to prevent the oviposition of other species.

### Acetylcholinesterase enzyme inhibition (LC_50_)

The inhibition of AChE enzyme was estimated by using the colorimetric assay kit (Biovision, Cairo, Egypt). The nanoformulation were dissolved in distilled water or AChE assay buffer to make different dilutions suitable to the kit requirements. Then add 10 µl of the diluted nanoformulation and positive control (Which pre-dissolved in proper solvent before) and placed 96 well plate. Then the solution of AChE Enzyme prepared as follow: 25-fold dilution of reconstituted AChE (i.e. dilute 2 μL of AChE with 48μL of AChE Assay Buffer) and mix well, then 10μL of Diluted AChE to each well containing tested compounds and positive and negative (solvent) control. Adjust the volume of each well to 160μL/well with AChE Assay Buffer. Mix well and incubate at room temperature for 10–15 min, protected from light. Then the samples absorbance was measured using BIOLINE ELISA READER and the wave length adjusted to be 450 nm.

### Statistical analysis

All statistical analysis was conducted with IBM SPSS Statistics Version 22 (IBM Corp. Armonk, NY, USA). The data of oxidative stress parameters were analyzed using non-parametric analysis (Kruskal Wallis test) and the median lethal concentration (LC_50_) of the NLC-MB-MT was analysed by the probit test. The graphs were prepared using Microsoft Excel Version 2016.

## Results

### Characterization of nanostructure lipid carrier incorporated monoterpenes and methylene blue (NLC-MB-MT)

#### Particle size and distribution by dynamic light scattering (DLS)

**Figure 1:**
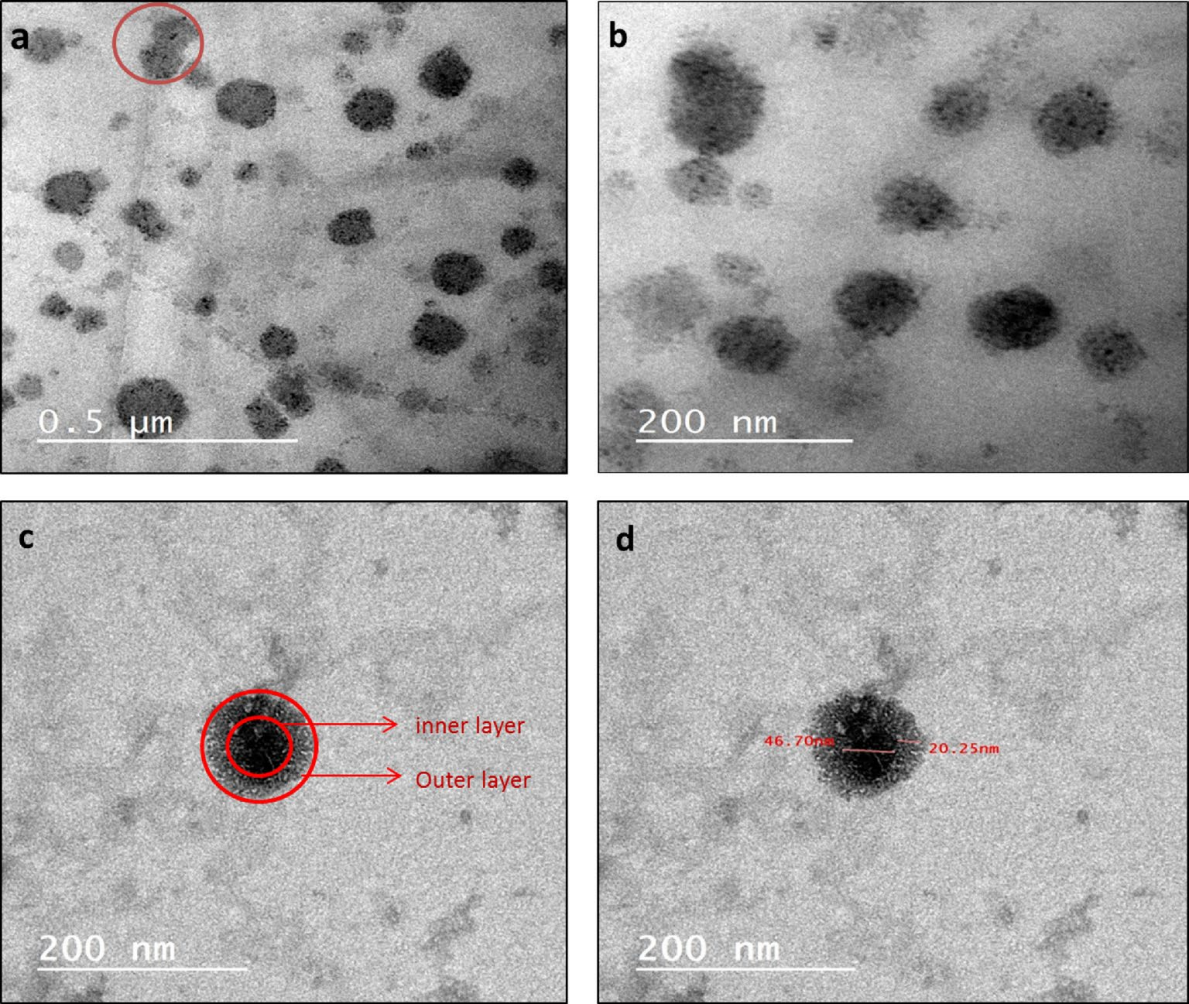
Internal structure by TEM of the prepared NLC-MB-MT.

The prepared NLC-MB-MT presented unique size measurements. The dynamic light scattering measurement of the prepared NLC-MB-MT (after 7 days) showed average size diameter of 73.23 nm and 69.38 nm respectively for zero-day and 7-day preparation indicating as clarified in Fig. 1S and Table 1.

**Table 1:** Average size, polydispersity index and zetapotential of the prepared NLC-MB-MT.

Time of preparation	DLS	PDI	Zeta potential
Zero day	73.23 ± 8 nm	0.096 ± 0.011	− 27.4 ± 0.3 mV
7 days later	69.38 ± 5 nm	0.092 ± 0.008	− 25 ± 0.7 mV

### Polydispersity index and homogeneity

According to Fig. 1S and Table1 the polydispersity index of the NLC-MB-MT equal to 0.096 and 0.092 for zero-day and 7-day preparation, respectively. This result indicated PDI value < 0.1 by the mean the size distribution was highly homogeneous and that is very close to be monomodal particle size distribution.

#### Zeta potential and charge

**Figure 2:**
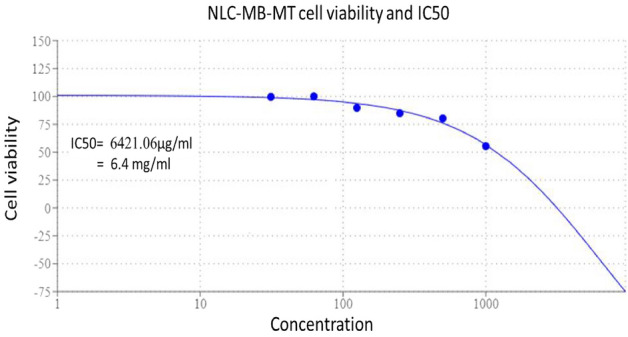
cytotoxic activity and IC_50_ evolution of NLC-MB-MT nanoparticles on WI38 normal cell line.

After 7 days preparation the value of zeta potential of the prepared NLC-MB-MT nanoformulation exhibited negative sign as net charge density as clarified in Fig. 2S and Table 1 with high numerical value of − 27.4 mV and − 25 mV, respectively at zero-day and 7-day preparation indicating very good physical stability behavior and low tendency to aggregation.

#### Internal structure and particle morphology

The prepared NLC-MB-MT depicted regular spherical and semispherical particle shape in rage of size less than 200 nm as showed Fig. 1a and with the presence of negligible aggregation (marked with red cycle). Qualitatively, the fields in Fig. 1a and b presented particles in comparable and narrow size distribution also most of the nanoparticles in range of 66 nm. Furthermore, the encapsulation of lipid layer as outer layer and target compounds (MB, carvacrol and citronellal) on the inner layer that was clearly showed in Fig. 1c and d.

### In-vitro cytotoxicity effect of the NLC-MB-MT nanoformulation on normal human cell lines (MTT assay)

The fibroblast human lung cell lines WI38 was chosen randomly as preliminary test to evaluate the cytotoxicity effect of the NLC-MB-MT nanoformulation. The lower IC_50_ concentration indicated the more potent drug activity and that is not favorable in the cytotoxic activity evaluation using WI38 cell lines as it is indicating more sensitivity towards the cell lines and consequently the human tissue. After 48 h incubation the half maximal inhibitory concentration that inhibits 50% of the cells IC_50_ equal to 6.4 mg/mL as showed in Table 2 and Fig. 2. Such result is very good relative to chemical pesticides as 2,4-phenoxy acetic acid (IC_50_ after 72 h = 115 ± 4.39 µg/mL)^59^.

**Table 2:** MTT assay and cytotoxic effect of NLC-MB-MT on normal fibroblast cell line (WI38).

Conc. (µg/mL)	Mean of OD ± SE	Viability (%)	Toxicity (%)
Untreated	0.79 ± 0.00	100	0
1000	0.44 ± 0.00	55.47	44.53
500	0.63 ± 0.01	80.28	19.72
250	0.67 ± 0.01	84.87	15.13
125	0.70 ± 0.01	89.77	10.23
62.5	0.79 ± 0.00	100	0
31.25	0.78 ± 0.00	99.55	0.45

### Molecular docking of acetylcholine esterase enzyme

**Figure 3:**
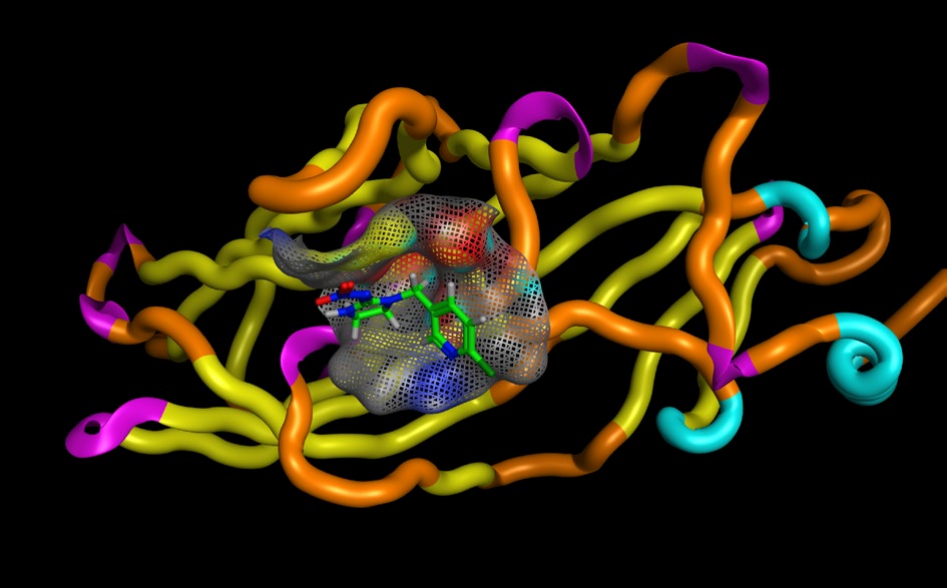
Three dimensional receptor positioning of Chain A and the active site incorporated imidacloprid co-crystallized ligand of 2ZJU protein.

**Figure 4:**
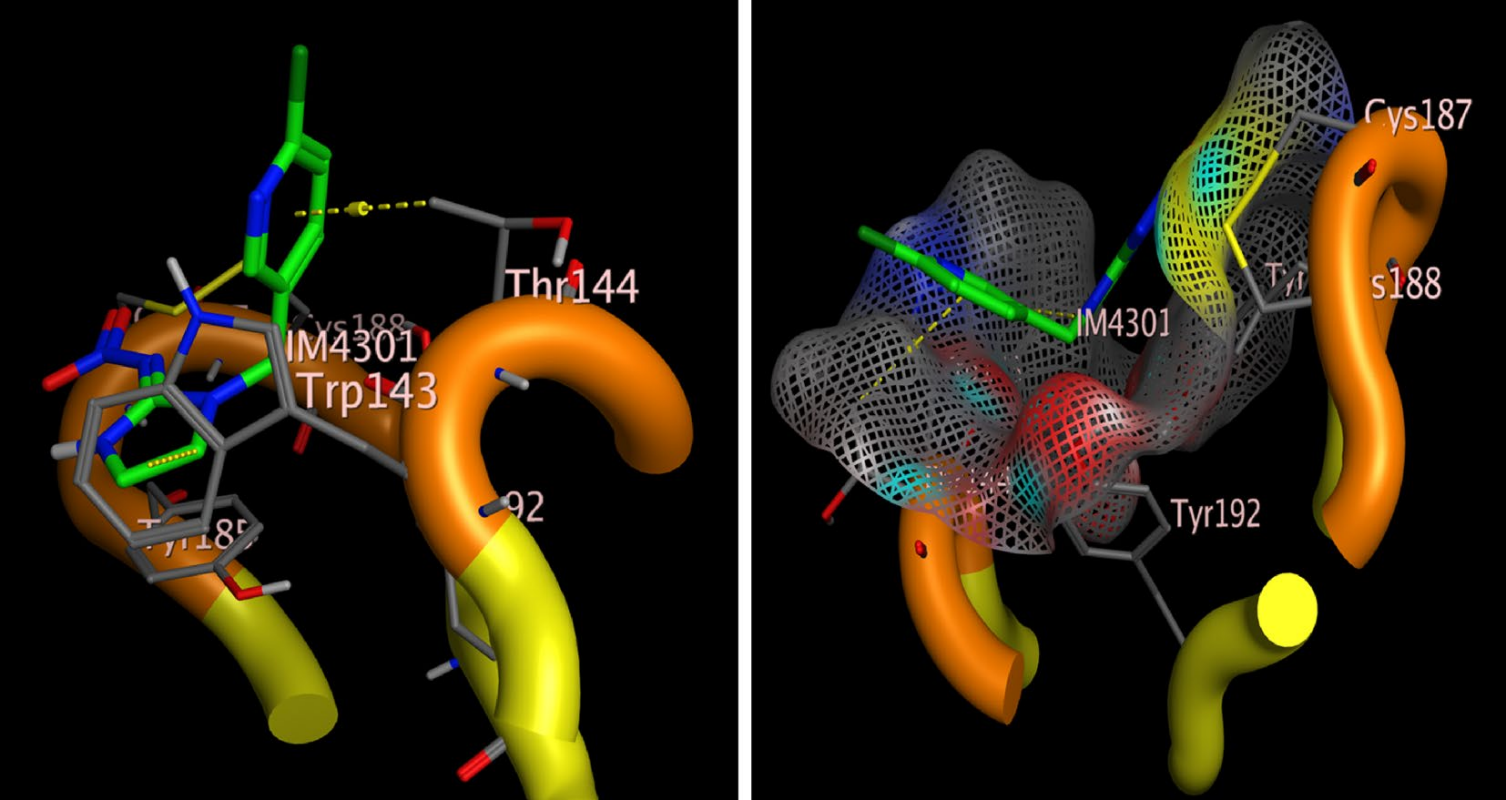
Three dimensional receptor positioning of self-Docking of the (imidacloprid) co-crystallized ligand interior 2ZJU protein.

**Figure 5:**
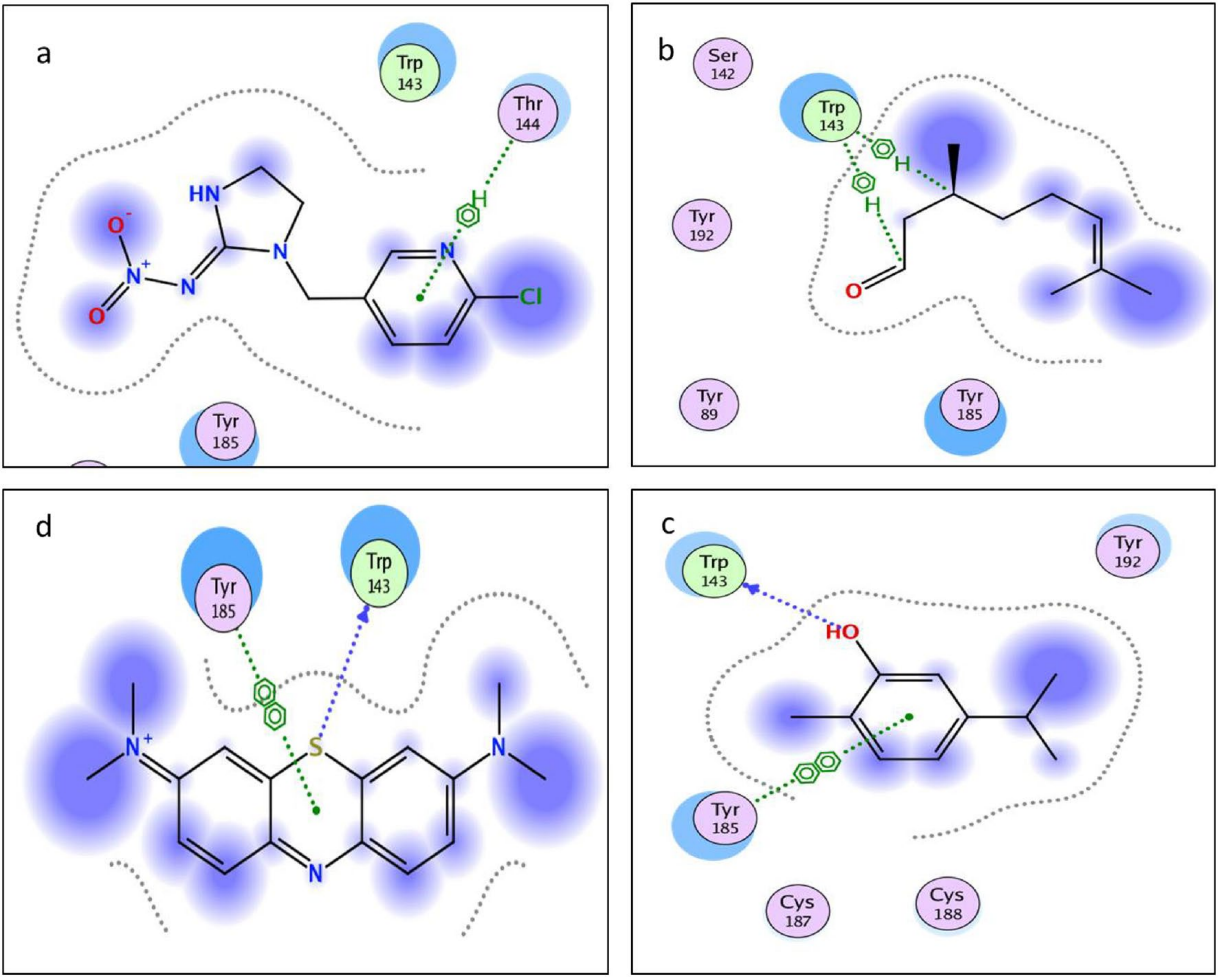
Two dimensional interactions of the co-crystallized ligand (**a**) Imidacloprid and target compounds (**b**) citronellal, (**c**) carvacrol and (**d**) methylene blue interior 2ZJU protein active site.

Molecular docking study was performed within the active site pocket of 2ZJU protein crystalized with imidacloprid insecticide Fig. 3. Comparing to the co-crystallized ligand (imidacloprid) binding interactions showed in Fig. 4, docking results revealed good binding affinity towards all the tested compounds (tested ligands) with accepted binding energies and RMSD values as shown in Table 3 and Fig. 5.

**Table 3:** Docking results of compounds Imidacloprid, carvacrol, citronellal, and methylene blue interior 2ZJU protein active site.

Compound	Interaction	Type	Distance	Score	Rmsd
Imidacloprid	Thr144–benzene ring	Aromatic	–	–	–
Carvacrol	Trp143–OH	Hydrogen bond	1.11	− 3.93443322	1.08430552
	Tyr185–benzene ring	Aromatic	–		
Citronellal	Trp143–C	Aromatic	–	− 4.06693888	0.901263118
	Trp143–C=O	Aromatic	–		
Methylene blue	Tro143–S	Hydrogen bond	1.65	− 4.5026083	0.718820691
	Tyr185–benzene ring	Aromatic	–

### Effect of NLC-MB-MT on the hydrogen peroxide level (H_2_O_2_)

**Figure 6:**
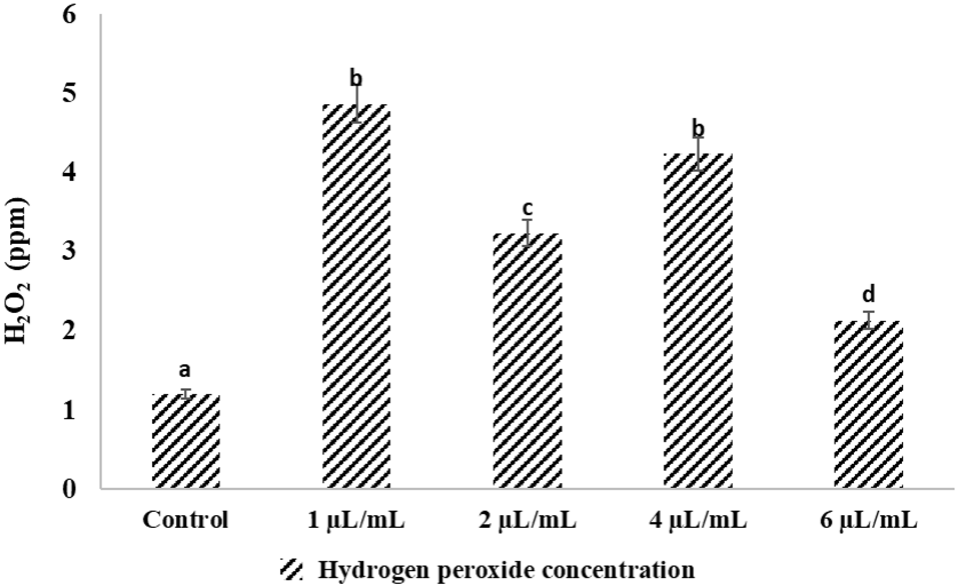
Effects of NLC-MB-MT nanoformulation on hydrogen Peroxide level in mosquito. Bars marked with the same small letters showed non-significant difference between different concentration treatments (P > 0.05).

The larval tissue homogenates from the control groups contained the least amount of H_2_O_2_. The fluctuation increasing pattern of the concentration of H_2_O_2_ served as evidence of the impact of the various doses of NLC-MB-MT injection. The concentration of H_2_O_2_ was found to be maximum at 1 µL/mL, then it decreased to 2 µL/mL, fluctuated by increasing at 4 µL/mL, and eventually decreased to 6 µL/mL, till it virtually approached the control groups at 12 h post injection, Fig. 6. Experimental larvae showed significant physiological changes in the activities of the investigated H_2_O_2_ parameters after stressor administration, as compared to pertinent controls.

### Effect of NLC-MB-MT on the protein carbonyl amounts

**Figure 7:**
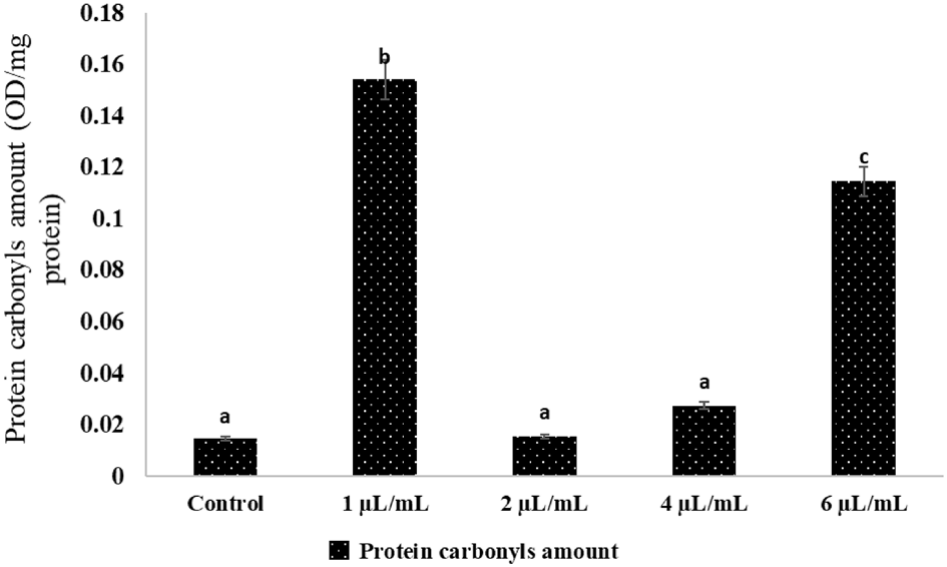
Effects of NLC-MB-MT nanoformulation on the total protein carbonyls amount of mosquito larvae. Bars marked with the same small letters showed no significant difference between different concentration treatments (P > 0.05).

The protein carbonyl content of the *Cx. pipiens* larvae was significantly affected by the NLC-MB-MT nanoformulation treatment at 12 h after injection, particularly at 1 µL/mL where it reached the highest level (0.15 OD/mg), followed by 0.12 OD/mg at 6 µL/mL, before total protein abruptly decreased to 0.02 OD/mg at 4 µL/mL, and continued until reaching the control at 2 µL/mL recording 0.01 OD/mg. The total protein was dramatically reduced after reaching a level that was higher than the controls 12 h after injection as shown in Fig. 7.

### Effect of NLC-MB-MT on the SOD activity and Ascorbic acid concentration

**Figure 8:**
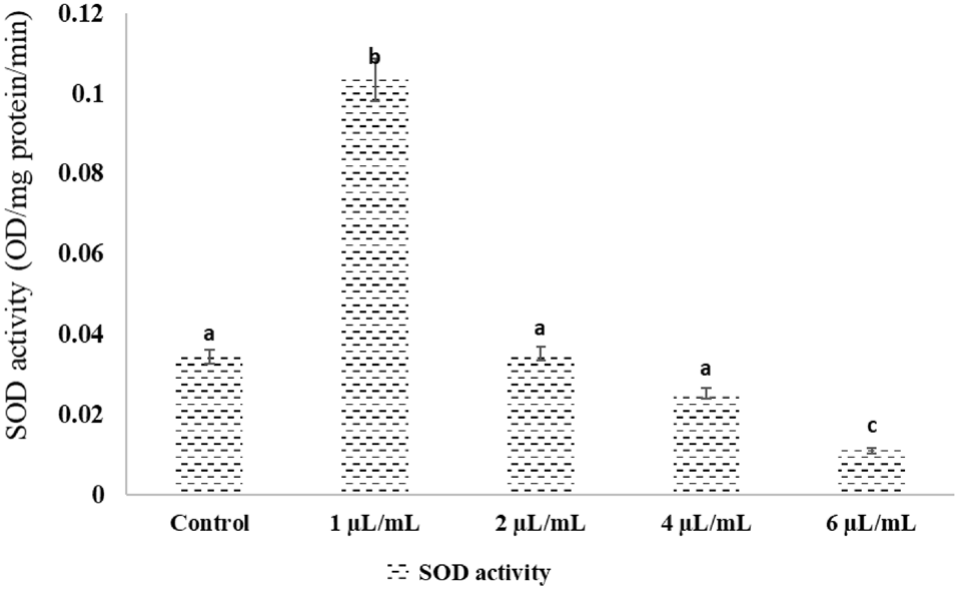
Effects of NLC-MB-MT nanoformulation on the SOD activity of mosquito larvae. Bars marked with the same small letters showed no significant difference between different concentration treatments (P > 0.05).

**Figure 9:**
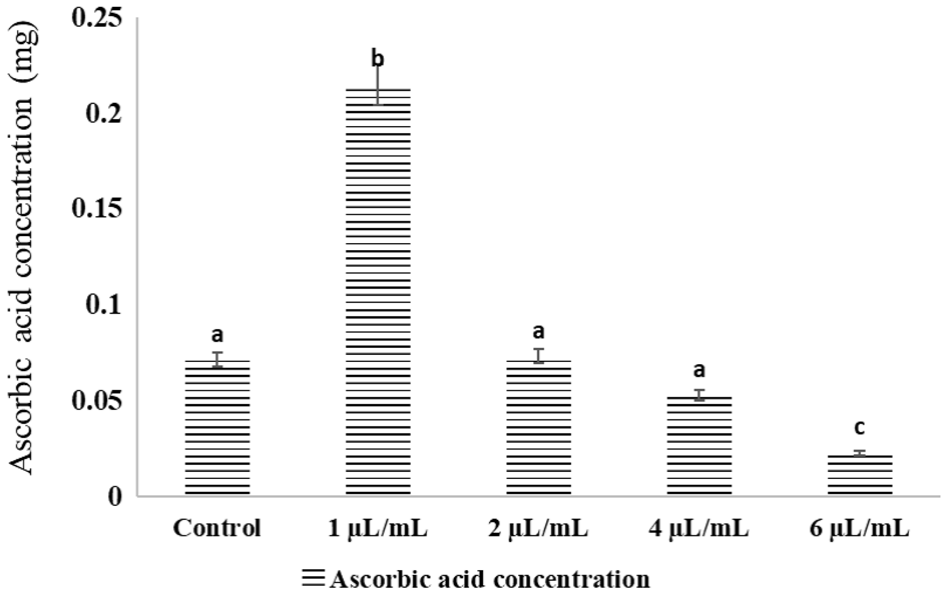
Effects of NLC-MB-MT nanoformulation on the ascorbic acid concentration of mosquito larvae. Bars marked with the same small letters showed no significant difference between different concentration treatments (P > 0.05).

There were non-significant differences (P > 0.05) in both SOD activity and ascorbic acid concentration of mosquito larvae after treatment with NLC-MB-MT nanoformulation concentrations (1, 2, 4, 6 µL/mL) after 12 h post injection compared to the control groups for the two physiological measures, Figs. 8 and 9. While the maximum levels for SOD and ascorbic acid were observed at 1 µL/mL.

### Acetylcholinesterase enzyme inhibition (LC_50_)

**Figure 10:**
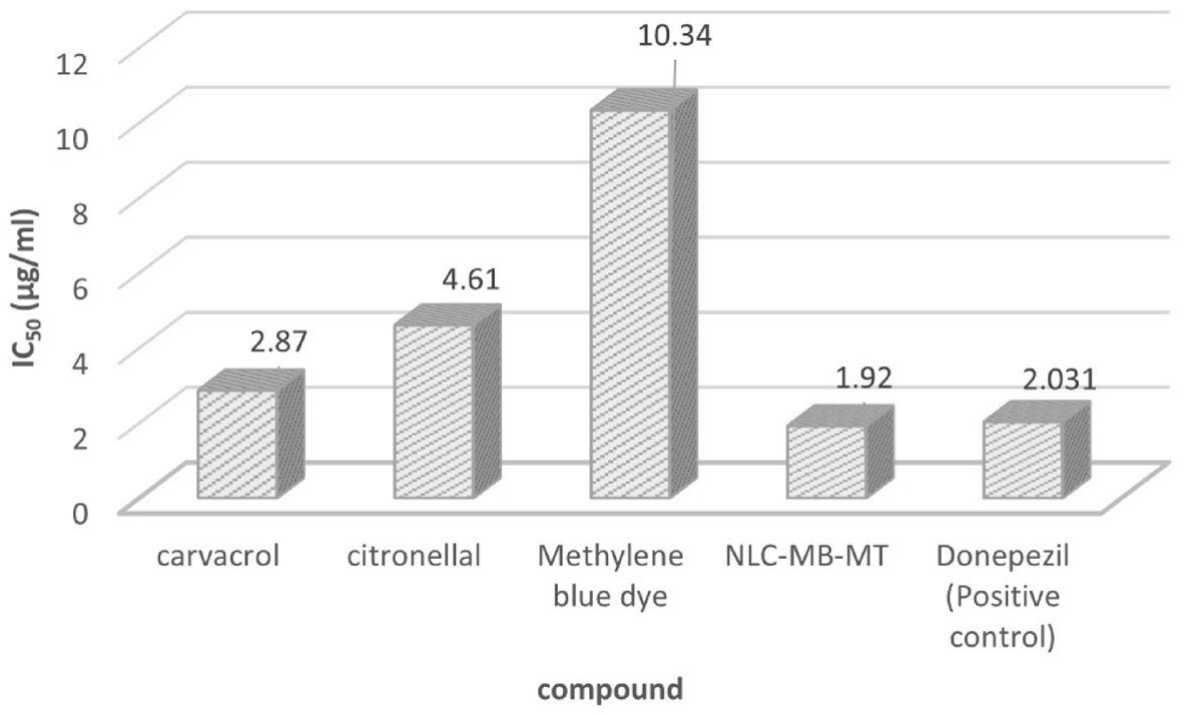
acetylcholinesterase enzyme inhibition of the tested compounds.

Acetylcholinesterase enzyme inhibition using colorimetric kite and the values of LC_50_ listed in Table 4 and Fig. 10. Each single compound revealed 10.34, 4.61, 2.87 and 1.92 µg/mL for MB dye, citronellal, carvacrol and NLC-MB-MT, respectively against the positive control donepezil (2.031 µg/mL).

**Table 4:** Acetylcholinesterase activity in *Cx. pipiens* exposed to different compounds.

Compound	Acetylcholinesterase	
	LC_50_ (µg/mL)	SD
Carvacrol	2.87	± 0.19
Citronellal	4.61	± 0.23
Methylene blue dye	10.34	± 0.34
NLC-MB-MT	1.92	± 0.13
Donepezil (positive control)	2.031	± 0.11

### Interaction and correlations between studied enzymes

The levels of total antioxidant capacity at 12 h PI in the tested tissues were highly positively correlated with the evaluated physiological parameters. The levels of H_2_O_2_ and the amount of protein carbonyl were typically reported to be in the previous positive pattern at 12 h post-PI. In a nutshell, untreated larvae raised in a lab under normal settings (the negative control) had considerably lower levels of oxidative stress markers in their tissues than those injected with various NLC-MB-MT nanoformulation dye dosages Figs. 8 and 9. However, as can be shown in Figs. 8 and 9, the values of ascorbic acid and SOD activity were close to those of the control groups.

### Larvicidal toxicity analysis

We measured the larvicidal effect of NLC-MB-MT on the fourth instar *Cx. pipiens* with different concentrations (0.5, 0.25, 0.125 and 0.0625 µL/mL. The toxicologically results of the study showed that the NLC-MB-MT nanoformulation has high virulence against the mosquito larvae, initiating higher mortality rate with LC_50_ of 0.1780 and 2.321 µL/mL after 24 and 48 h, Table 5. Subsequent to stressor application compared to relevant controls, experimental larvae exhibited significant physiological changes in activities of the tested parameters in response to stressor challenge, as can be seen.

**Table 5:** LC_50_ values of fourth instar of *Cx. pipiens* exposed to NLC-MB-MT nanoformulation.

Material	Time	LC_50_ (µL/mL)	Equation	R2
NLC-MB-MT	24 h	1.780	y = − 0.9268x + 6.6495	0.8909
	48 h	2.321	y = − 0.8969x + 6.5658	0.8691

## Discussion

The quality of any nanoparticles mainly determined by measuring their size and morphology, the particle size could be measured using DLS technique which measures the correlations (automatic correlation function) of the time-dependent fluctuations of the light scattered made by the nanoparticle's suspension which is governed by their Brownian motion^60^. By fitting the autocorrelation function, the diffusion coefficient determined and then the Stocks-Einstein is used to calculate the radius and consequently the hydrodynamic diameter. The present results is one of the smallest size of nanostructured lipid carrier with average size diameter of 69.38 nm, as usual NLC have particle size within range from 200 to 400 nm^61^ and some other NLC have extended size up to 1000 nm^62^. Preparation of smaller size than 200 nm required excessive increasing in the surfactant concentration however, producing NLC smaller than 100 nm is attracting particular interest as a result of their superior to penetrate cellular barrier especially, in biological system^63^. PDI is very important parameter indicating homogeneity and stability of the synthesized nanoparticles. PDI for width sizePDI values for wide-size distribution is ranging from 0 to 1, smaller values of PDI = 0 indicates complete monodispears size distribution (monomodal), while PDI > 0.5 referees to polydisprese or bread size distribution^50,64^.

The analysis of DLS in addition polydispersity index assumes the measured nanoparticles objects are of ideal spherical shapes. As a result of the fact of the particle size is exponential function-dependent to the scattering intensity, that means very small number of large molecules often masks the small ones, that will introduce misleading results and makes the device will not differentiate between small aggregated particles and larger ones^65^. Decreased of PDI values in case of NLC-MB-MT nanoformulation confirmed that the particle size distribution was best to be described with "narrow- size", that means all the particle size have comparable sizes and consequently wide size variation is limited.

The preparation of narrow size distributed nanoparticle is a magnificent challenging but really the stability factor should be taken into consideration, zeta potential is a physical property exhibited by any particle in a suspension comes from the idea of most colloidal system in aqueous medium carry an electric charge. Knowing the value of zeta potential could reduce the number of trials needed to design stable nanoformulations. Zeta potential of the prepared NLC-MB-MT nanoformulation exhibited negative sign not only due to the acidic lipid contents (oleic and stearic acid) but also to the negatively charged particles of the phenolic carvacrol and the polar aldehyde citronellal mono terpenes in addition the slightly acid MB dye. As a reason of the accumulation of the negative charges generates sufficient high repulsion force between the particles that makes the dispersion resist the flocculation or coagulation and the colloidal system will be stable. As increasing magnitude of the z.p values indicated more repulsion force and enhanced stability^66^. The best value of zeta potential are >  + 30 and <  − 30 mV^67^. Zeta potential of the nanoformulation NLC-MB-MT was found to be − 25 mV after 7 days preparation, this value is not the best zeta potential ever but it is indicating very good physical stability^68^ and high repulsive forces between the colloidal particles.

The internal morphology of the nanoparticles is best to be described by the transmission electron microscope as it is support us with in-situ vision of the particle shapes and homogeneity^69^. The prepared NLC-MB-MT presented spherical and semispherical particle varied in their regularity in comparable rage of size less than 200 nm. Some of negligible aggregation presented, in-consistent with the outcomes obtained from zeta potential results. Qualitatively, Most of the synthesized nps presented comparable particle sizes with narrow size variation in addition most of the nanoparticles in range of 66 nm. The DLS, Zeta potential inconsistence with TEM analysis, confirmed the synthesis of stable narrow size-distributed nanostructure lipid carrier.

To determine how the co-delivered compounds could bind to the protein active site, docking study was done using chain A. From Table 1, all one of the tested compounds could bind to the active site with two different bonds varied from hydrogen bonding to hydrophobic bonds (aromatic), for example both carvacrol and MB could bind with one hydrogen bond and another aromatic interaction, whereas citronellal bind with two aromatic interactions. Comparing the docking results of the three ligands under investigation to the imidacloprid as co-crystalized ligand, the imidacloprid showed one aromatic interaction with the aminoacid (Thr185), meanwhile citronellal and carvacrol have interactions with the amino acids (Trp143) and (Thr185) with Root Mean Square Deviation (RMSD) values 0.901263118and 1.08430552, respectively. Such accepted RMSD values^52^ values with low binding energy (− 4.06693888and − 3.93443322, respectively) confirmed that all the three compounds have successive binding modes more effective than imidacloprid to the vicinity of 2ZJU protein active site with acceptable binding energy. The use of bioinformatics is crucial to reduce the probabilities, in this study and according to the results of the molecular docking study which confirmed that using carvacrol, citronellal and MB will be effective and each one of them could replace the Imidacloprid with some types of accepted interaction and accepted binding modes. AChE inhibition assay were done for each separate compound in addition to the nanoformulation which contains merging the three active ingredients. The synthesized nps showed very good enzyme inhibition with LC_50_ = 1.92 µg/ml while the positive control, Donepezil, LC_50_ = 2.031 µg/ml. Similar studies were done to evaluate imidacloprid as synthetic insecticide the value of AChE enzyme inhibition LC_50_ was 0.0024 µM^70^ which equivalent to 0.6135 µg/ml. so the enzyme inhibition made by NLC-MB-MT supported with safety studied by treatment with normal cell line is very interesting if compered to the positive controls Donepezil and imidacloprid regarding to both enzyme inhibition and their safety drawbacks^71^.

The lower LC_50_ concentration of the NLC-MB-MT nanoformulation indicated the more potent drug activity and that is not favorable in the cytotoxic activity evaluation using WI38 cell lines. Those results are very good relative to chemical pesticides as 2,4-phenoxy acetic acid (LC_50_ after 72 h = 115 ± 4.39 µg/mL)^59^.

There is limited data on the effects of NLC-MB-MT nanoformulation on the parameters of oxidative stress, namely total antioxidant ability and reducing power. This information was taken into consideration while interpreting the findings of this experiment. Elevated quantities of detoxifying and antioxidant enzymes are produced by insects as a result of their defense mechanisms against certain insecticides^72–74^. By focusing on the conversion of superoxide anion radicals (O_2_⋅−) into oxygen and H_2_O_2_, these findings provide an explanation of how SOD enzymes function^75^. The highly created H_2_O_2_ concentration is converted by CAT enzymes to oxygen and water^76,77^. Hydroxyl radical production causes protein oxidation and denaturation when the rate of O_2_ anions or H_2_O_2_ breakdown is insufficient.

Oxidative stress parameters as a biomonitoring of pesticides applications were evaluated in various studies^78,79^. Due to a number of foreign and internal causes, (including the treatment of NLC-MB-MT nanoformulation) increases the formation of H_2_O_2_ in living things, the levels of ROS generation are raised. Similar to previous findings, the current findings demonstrated a notable raise in H_2_O_2_ generation rate nearly in each concentration of NLC-MB-MT. After 12 h post injection, the treatment of *Cx. pipiens* larvae causes an increase in ROS generation, particularly H_2_O_2_. The findings supported by Fridovich^80^ theory that endogenous toxicant emit ROS that reveal elevated amounts of H_2_O_2_ when they enter an insect's body^81^. H_2_O_2_ damages cell membranes by oxidizing the proteins that make up the membranes (protein oxidation)^39,82^. The observed drop in total protein may be the result of protein breakdown into free amino acids brought on by the stress of the treatment. This may indicate that the insect was attempting to physiologically counteract the effects of the H_2_O_2_ by producing defense proteins^83^.

The ROS accumulate as a result of oxidative stress when there is an imbalance between their synthesis and elimination^84,85^. As a result of oxidative stress, substances like DNA single strand breaks are all examples of damage to macromolecules^44,86^, protein carbonylation^2,87^ and lipids suffer oxidative damage^73,88^. The cell macromolecular processes that produce oxidative damage can be minimized by an antioxidant defense system that includes both enzymatic and non-enzymatic mechanisms. Differences in biotic and abiotic circumstances, as well as harsh environmental variables including bacterial, fungal, and viral infections, insecticide, low or high temperature, among others, govern oxidative damage among insect species. Super oxide dismutases (SODs) are frequent enzymes in living things that become active when there is oxygen present. According to Łukasik et al.^42^, increasing oxidative stress caused a considerable decrease in ascorbate, the main non-enzymatic antioxidant that, along with ascorbate peroxidase which helps aphids get rid of harmful H_2_O_2_. These enzymes help O_2_ be transformed into oxygen and H_2_O_2_. The ROS levels are regulated by SOD enzymes. However, in addition to the various concentrations, there was a reduction in Ascorbic acid and SOD activity in the mosquito larvae. Likewise, compared to the control, as shown in* Spodoptera exigua* larvae exposed to high humidity had significantly decreased SOD, CAT, and peroxidase activity^89^. These findings shed light on the various ways by which high insect mortality results in depleted antioxidant systems. The potential for using the antioxidant response of mosquito larvae or other oxidative stress indicators as a marker of NLC-MB-MT nanoformulationwas also evaluated. Additionally, a new approach of possible application of NLC-MB-MT nanoformulation as a disruptor of the mosquito larval tissues was presented.

## Conclusion

Finally, our results demonstrate that these toxic effects of NLC-MB-MT were evidenced by significant changes in the activity of defensive enzymes. The present study addressed the use of natural monoterpenes and MB as co-delivery system incorporated into NLC-MB-MT as insect control. The reactive oxidative stress led to some biochemical changes in the mosquito larval tissues dependent on model of NLC-MB-MT nanoformulation application. The cytotoxic evaluation against WI38 cell line give initiative cytotoxic results so, it may help in a further investigation of the NLC-MB-MT nanoformulation toxicity in field application. Further studies on the pharmacological characterization of insect NLC-MB-MT nanoformulation may help us to develop new specific insecticides for pest management.

## Supplementary Information

Supplementary Figures.

## Data Availability

This article presents all data created or analysed throughout the investigation.
