# Supplementary material for: Effect of nanostructure lipid carrier of methylene blue and monoterpenes as enzymes inhibitor for Culex pipiens
Source: Sci Rep. 2023 Aug 2;13:12522. doi: 10.1038/s41598-023-39385-y (PMC10397322; doi:10.1038/s41598-023-39385-y)
Supplement: Supplementary file 1 — Supplementary Figures. [file 41598_2023_39385_MOESM1_ESM.doc]

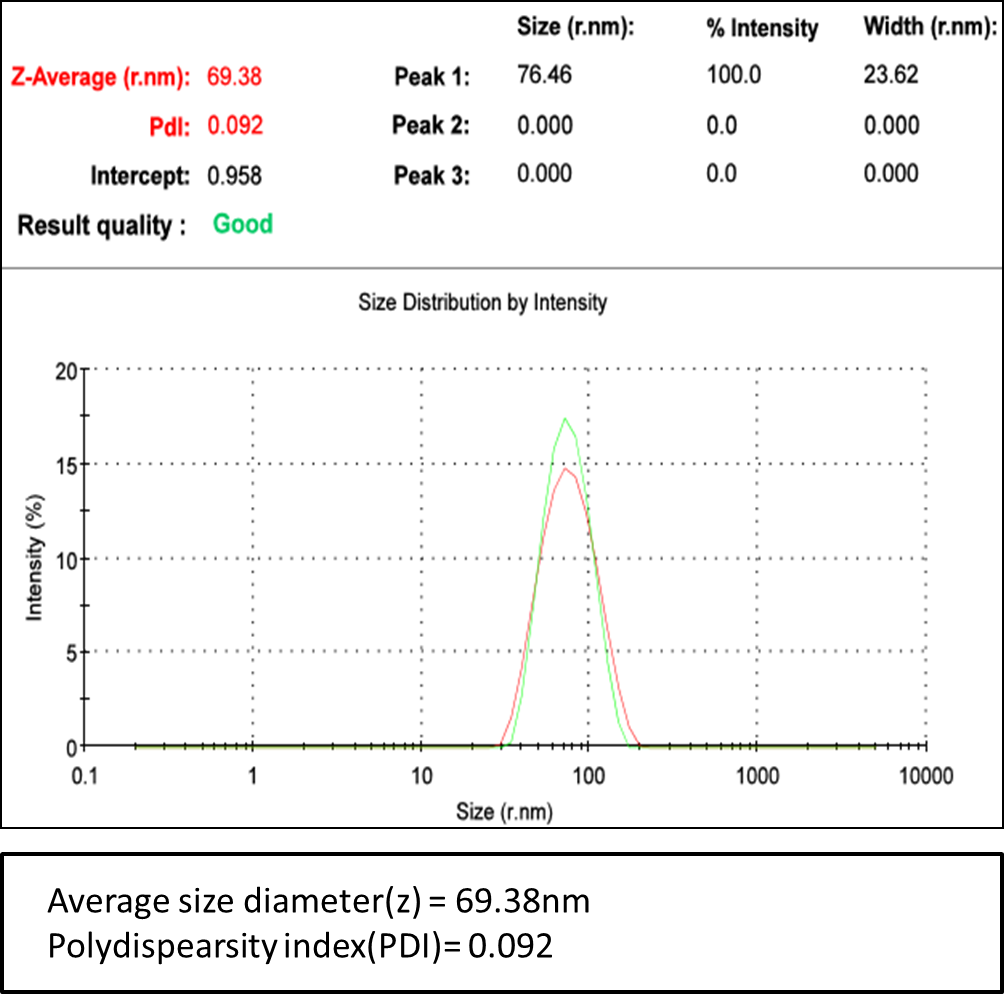


# Figure 1S: Average size distribution and polydispersity index of the prepared NLC-MB-MT

#
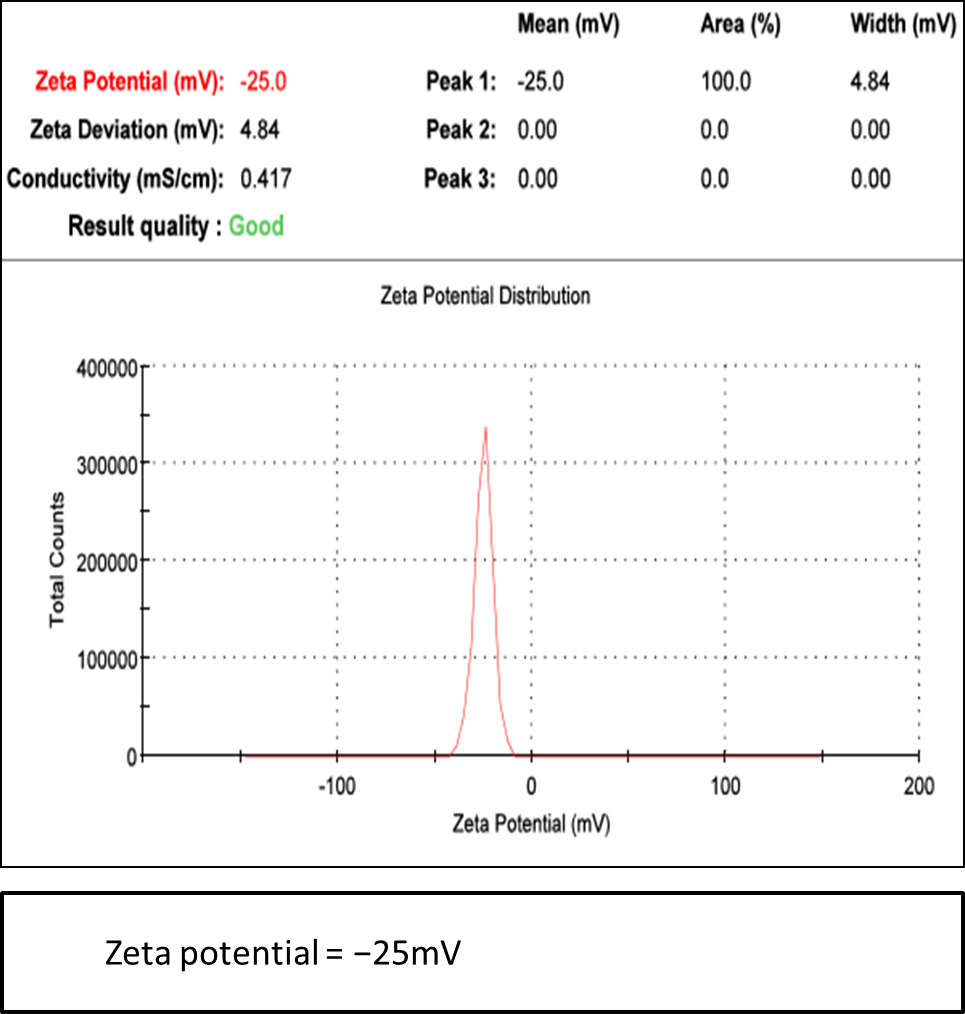


# Figure 2S: Zeta potential of the prepared NLC-MB-MT
